# Changes in appetite related gut hormones in intensive care unit patients: a pilot cohort study

**DOI:** 10.1186/cc3957

**Published:** 2005-12-23

**Authors:** Mohsen Nematy, Jacqui E O'Flynn, Liesl Wandrag, Audrey E Brynes, Stephen J Brett, Michael Patterson, Mohammad A Ghatei, Stephen R Bloom, Gary S Frost

**Affiliations:** 1 Phd Student, Nutrition and Dietetic Research Group, Imperial College, Hammersmith Hospitals NHS Trust, Du Cane Road, London W12 0HS, UK; 2 Senior Dietician, Nutrition and Dietetic Research Group, Imperial College, Hammersmith Hospitals NHS Trust, Du Cane Road, London W12 0HS, UK; 3 Chief Research Dietician, Honorary Lecturer Imperial College, Nutrition and Dietetic Research Group, Imperial College, Hammersmith Hospitals NHS Trust, Du Cane Road, London W12 0HS, UK; 4 ICU Consultant, Division of Surgery, Anaesthetics and Intensive Care, Imperial College, Hammersmith Hospitals NHS Trust, Du Cane Road, London W12 0HS, UK; 5 Graduate student, Department of Metabolic Medicine, Imperial College, Hammersmith Hospitals NHS Trust, London W12 0NN, UK; 6 Professor of Metabolic Medicine, Department of Metabolic Medicine, Imperial College, Hammersmith Hospitals NHS Trust, London W12 0NN, UK; 7 Professor of Nutrition, Department of Metabolic Medicine, Imperial College, Hammersmith Hospitals NHS Trust, London W12 0NN, UK; 8 Professor of Nutrition, Nutrition and Dietetic Research Group, Imperial College, Hammersmith Hospitals NHS Trust, Du Cane Road, London W12 0HS, UK

## Abstract

**Introduction:**

The nutritional status of patients in the intensive care unit (ICU) appears to decline not only during their stay in the ICU but also after discharge from the ICU. Recent evidence suggests that gut released peptides, such as ghrelin and peptide YY (PYY) regulate the initiation and termination of meals and could play a role in the altered eating behaviour of sick patients. The aim of this study was to assess the patterns of ghrelin and PYY levels during the stay of ICU patients in hospital.

**Methods:**

Sixteen ICU patients (60 ± 4.7 years, body mass index (BMI) 28.1 ± 1.7 kg/m^2 ^(mean ± standard error of the mean)) underwent fasting blood sample collections on days 1, 3, 5, 14, 21 and 28 of their stay at Hammersmith and Charing Cross Hospitals. Changes in appetite and biochemical and anthropometric markers of nutritional status were recorded. A comparison was made to a group of 36 healthy volunteers matched for age and BMI (54.3 ± 2.9 years, *p *= 0.3; BMI 25.8 ± 0.8 kg/m^2 ^*p *= 0.2).

**Results:**

Compared to healthy subjects, ICU patients exhibited a significantly lower level of ghrelin (day one 297.8 ± 76.3 versus 827.2 ± 78.7 pmol/l, *p *< 0.001) during their stay in the ICU. This tended to rise to the normal level during the last three weeks of hospital stay. Conversely, ICU patients showed a significantly higher level of PYY (day one 31.5 ± 9.6 versus 11.3 ± 1.0 pmol/l, *p *< 0.05) throughout their stay in the ICU and on the ward, with a downward trend to the normal level during the last three weeks of stay.

**Conclusions:**

Results from our study show high levels of PYY and low levels of ghrelin in ICU patients compared to healthy controls. There appears to be a relationship between the level of these gut hormones and nutritional intake.

## Introduction

Impaired appetite is a common feature of illness. A recent review suggested that 10% to 40% of adult patients admitted to hospital exhibit some level of nutritional depletion [1], with much weight loss, occurring over the period of hospitalisation [2]. This is a matter of particular concern for certain categories of patients. Patients in intensive care units (ICUs) are a vulnerable group. One report suggested 55 out of 129 patients admitted to ICUs were already suffering from malnutrition [3]. Their nutritional status further declines during the intensive care and also after their ICU stay [3]. This study noted that despite 20 years of intense awareness, malnutrition was still highly prevalent in hospitalised patients and this continues to affect patients' outcomes. At present, nutritional supplements are used in most hospitals, but their effectiveness varies [4], probably due to the influence of poor appetite. The mechanism of poor nutritional intake in ICU patients remains unclear, but gastric myoneural inhibition and gastrointestinal hypomotility with delayed gastric emptying may be contributing factors.

Peptide hormones released from the gut, such as ghrelin and peptide YY (PYY), which stimulate and inhibit the appetite, respectively [5,6], might play a role in the altered eating behaviour of sick patients because the anorexia in sick hospitalised patients is often characterised by a premature feeling of fullness and loss of hunger. Ghrelin, a 28 amino acid peptide, is produced by the stomach and its level is highest in the fasting state, rising sharply before, and falling within one hour of, a meal [7]. Wren and colleagues [8] demonstrated that intravenous ghrelin infusion stimulates appetite and food intake potently in human and recent research showed that circulating ghrelin levels decreased in normal weight subjects after a meal [9]. Ghrelin, a newly discovered gut peptide is potentially an important new peripheral signal to the brain to stimulate food intake in human [8].

The levels of ghrelin and PYY in sick hospitalised patients are unknown. Peptide YY is a naturally occurring peptide that is released into the circulation by intestinal L-cells following food ingestion. PYY_3–36 _is the major form of metabolically active PYY in both the gut mucosal endocrine cells and the circulation. In human volunteers, exogenous infusion of PYY _3–36 _reduces food intake by 30% compared to placebo [10]. Recent work on patients with cardiac cachexia caused by severe pulmonary hypertension showed an exaggerated and early PYY response to a test meal when compared to control subjects [11]. Research so far has demonstrated that PYY physiologically inhibits appetite in human and suggests it is likely to be important in the everyday regulation of food intake.

The aim of this study was to investigate the concentration of ghrelin and PYY in patients during their stay in the ICU and, secondarily, the relationship between these levels and measures of appetite and food intake.

## Materials and methods

### Study subjects

This was a prospective study undertaken at Hammersmith and Charing Cross Hospitals, London. Local ethics committee approval was obtained for the enrolment of both patients and control subjects. Conscious patients with adequate mental capacity gave written informed consent prior to enrolment; for others, a close relative/partner gave written assent, with deferred patient consent being obtained during recovery. Patient refusal at this point resulted in complete withdrawal from the study.

The inclusion criteria were: male and female patients between the ages of 18 and 85 years who were anticipated to stay in the ICU for longer than three days in the opinion of the consultant. Exclusion criteria were: patients who were anticipated to die, or stay less than three days in the ICU; those who were known to be HIV or hepatitis B surface antigen positive; and patients who were already enrolled in a therapeutic study. The study was performed in accordance with the Declaration of Helsinki.

### ICU patients

Nutritional and medical data were collected from patients, charts, medical notes, dieticians and medical teams. Patients were followed clinically until discharge from hospital or death.

#### Visual analogue scale

As patients recovered from critical illness in intensive care they were asked to complete a visual analogue scale (VAS) for appetite [12]. This was repeated on the days that blood samples were collected throughout their stay. VAS questionnaires were not obtained during intensive care stay as patients were mainly fed nasogastrically or received parenteral nutrition, and were not alert enough to reply to questions. Some patients were never well enough to complete the VAS assessment.

#### Food intake

Food intake was estimated from food record charts completed at ward level. Nurses were given instruction on how to complete the intake charts. The nutritional content of the patients' food record charts and healthy subjects' three-day diet diary were calculated using computerised food tables in Dietplan5 (Forrest Hill Software Ltd, Sussex, UK).

#### Anthropometric measurements

Anthropometric indices, including triceps skinfold thickness, muscle arm circumference (MAC) and weight were assessed at the nearest point to ICU discharge [13,14]. The admission weight was recorded from the notes if it was known, otherwise it was recorded at the nearest point to ICU discharge. The demi-span was measured to calculate the height in order to compute body mass index (BMI) [15].

#### Blood sampling

Fasting blood samples were taken on days 1, 3, 5, 14, 21 and 28 of stay or date of discharge home. In the ICU, fasting blood samples were taken at 6 a.m. In the ward, fasting blood samples were taken between 7 and 8 a.m. before starting breakfast. Blood samples were centrifuged at 4°C; plasma was then separated, frozen immediately and stored at -20°C until analysis. Patients were followed after transfer to other wards, until day 28 or discharge home if earlier.

Results from albumin, total protein, C-reactive protein (CRP), and haemoglobin concentrations were extracted from the patient records.

#### Acute Physiology and Chronic Health Evaluation II

Acute Physiology and Chronic Health Evaluation (APACHE) II and risk of death scores were calculated to stratify illness severity on admission [16] and to anticipate prognosis.

### Control volunteers

Healthy control volunteers were recruited by advertisement at Hammersmith Hospital.

The inclusion criteria were; volunteers between the ages of 18 and 85 years. Exclusion criteria were: those subjects with a history of significant chronic diseases or muscle wasting diseases; smoking; substance abuse; pregnancy; medical or psychiatric illness; and those who were known to be HIV or hepatitis B surface antigen positive. An appetite questionnaire (visual analogue scale) was completed for control subjects; this was repeated on the days that blood samples were taken and weight was recorded. Control subjects also completed a three-day diet diary to determine their food intake. A tape and callipers were used on their arm to measure MAC and triceps skinfold thickness. Their height and weight were measured in order to calculate their BMI. Subjects were asked to fast from 10 p.m. on the night before each visit and to have only water to drink from midnight. One 10 ml fasting blood sample between 7.30 and 9.30 a.m. on days 1, 3 and 5 were taken from each volunteer.

### Gut peptides assays

Plasma PYY and ghrelin were measured using 'established in-house radioimmunoassays' as described previously [17,18].

### Column chromatography

To confirm ghrelin and PYY like immunoreactivity represented endogenous ghrelin or PYY and not non-specific interference, plasma samples were fractioned by Sephadex G-50 gel permeation chromatography [17,18]. The eluted fractions were assayed for ghrelin and PYY immunoreactivity.

### Statistical analysis

A power calculation based on PYY and ghrelin concentration from an interim analysis suggested a sample size of seven patients matched to seven controls (at a power of 95%) would be enough to show significant changes between patients and control subjects (*P *< 0.05). The data were analysed using SPSS 12.0 for windows (SPSS Science, Apache Software Foundation, Chicago, IL, USA). All data were checked for normality and presented as mean ± standard error of the mean (SEM). An independent two-tailed *t *test was performed to compare the PYY and ghrelin patterns between patients and control groups. Correlation analysis was performed using Pearson correlation coefficient.

## Results

**Figure 1:**
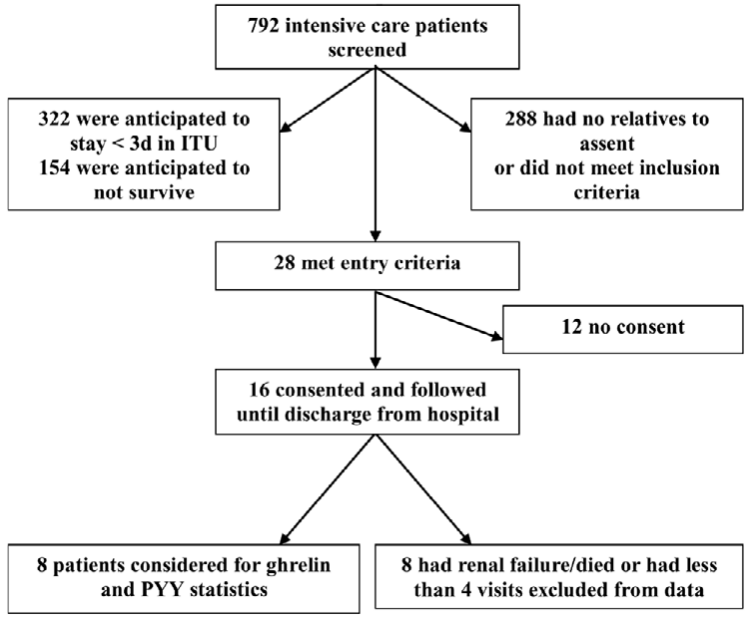
Flow of intensive care patients through the study. d, day; ITU, intensive therapy unit.

Sixteen ICU patients consented to enrolment and were followed until discharge. The study enrolment profile is shown in Figure 1. Four patients who required renal replacement therapy were excluded from the gut hormone final analysis. Four patients died during the study period and two patients had less than four blood samples (Table 1). Therefore, the sample size was reduced to eight eligible patients for ghrelin and PYY analysis (Table 1).

There was one assay failure for PYY so this reduced the sample size from eight to seven patients for PYY. We matched patients and controls for PYY and the number of controls was reduced accordingly.

### Demographics of patients and control volunteers

The comparative mean age and BMI of the patients and control groups are reported in Table 2. There were 36 healthy volunteers aged 54.3 ± 2.9 years (range 29 to 83 years) with a BMI of 25.8 ± 0.8 kg/m^2 ^(range19.8 to 40 kg/m^2^) recruited at Hammersmith Hospital (Table 2). Table 1 provides data on methods of feeding, APACHE II and risk of death scores, length of stay in the ICU and total hospital stay, diagnosis and reason of admission of patients to the ICU. Length of stay in the ICU was 12.9 ± 2.2 days (range 2 to 30 days) and total hospital stay was 40.3 ± 6.6 days (range 9 to 84 days). The results of the ghrelin and PYY assays from control subjects (little day to day variation) were grouped together as a pseudo normal range of mean ± SEM, with each subject contributing three data points.

### Gut hormones

#### PYY

**Figure 2:**
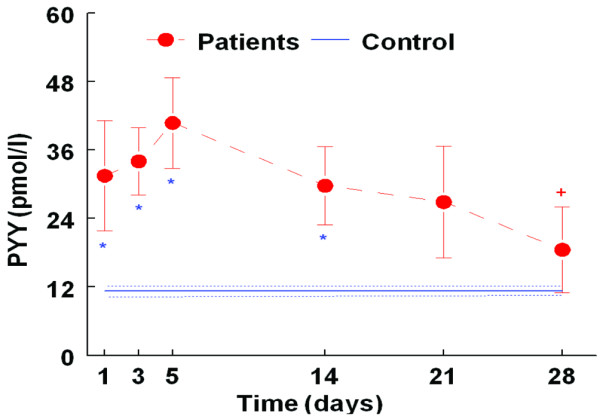
Pattern of plasma peptide (PYY; mean ± standard error of the mean) during intensive care unit (ICU) stay (n = 7 patients) compared with healthy age and body mass index matched control group (n = 31). Filled circles, ICU patients; solid line, control group; doted line, error bar in control group; * *p *< 0.05 for patients versus controls. There was no significant difference between patients and control subjects on day 21 and 28. ^+^*p *< 0.05 for patient day 3 and 5 versus patient day 28.

Fasting PYY levels were significantly higher in the ICU patients compared to the control group, as can be seen in Figure 2. Over the period of admission, however, PYY levels significantly fell; at days 21 and 28 there was no significant difference between the ICU group and the healthy controls. There was no significant difference between the initial plasma level of PYY in patients and the one prior to discharge due to a large variation of the level on day one (SEM = 9.6; Table 3). However, there were significant differences between the plasma level of PYY on days three and five of admission and that prior to discharge (34 ± 5.9 and 40.7 ± 7.9 versus 22.3 ± 6.3, *p *< 0.05 and *p *= 0.02, respectively; Table 3).

Renal failure is known to be associated with high levels of PYY, and it is currently unclear whether this represents the active PYY_3–36 _[19]. Of 16 patients, four had renal failure and significantly higher PYY levels than those patients that did not have renal failure (day 3 in ICU, 62.2 ± 5.2 versus 34.0 ± 5.9 pmol/l, *p *< 0.05). We defined renal failure here as dialysis dependency or chronic renal failure; these four patients received dialysis in ICU and were removed from subsequent analysis. A significant difference in PYY levels between patients and the control group is still clearly apparent, even with the removal of data for the four renal failure patients (Figure 2).

#### Ghrelin

**Figure 3:**
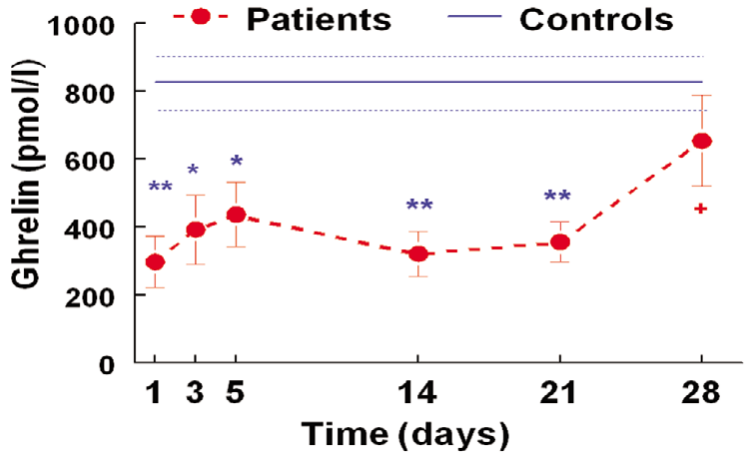
Pattern of plasma ghrelin (mean ± standard error of the mean) during intensive care unit (ICU) stay (n = 8 patients) compared with healthy age and body mass index matched control group (n = 36). Filled circles, ICU patients; solid line, control group; doted line, error bar in control group; * *p *< 0.05. ** *p *< 0.001 patients versus controls. There was no significant difference between patients and control subjects on day 21 and 28. ^+^*p *<*0.05 *for patient day 1 versus patient day 28.

Figure 3 shows that fasting ghrelin levels were significantly lower in the ICU patients compared with the control group during the first three weeks of stay; however, this difference disappeared over the fourth week of stay. There was a significant difference between initial levels of ghrelin and that prior to discharge (Table 3). As mentioned above, the four patients who had renal failure were removed from the subsequent analysis. Among the ICU patients, there was no significant difference between men and women. There was no significant difference in PYY and ghrelin concentrations between actual feeding groups (oral, nasogastric and parenteral nutrition), although this was an underpowered observation (data not shown).

#### C-reactive protein

**Figure 4:**
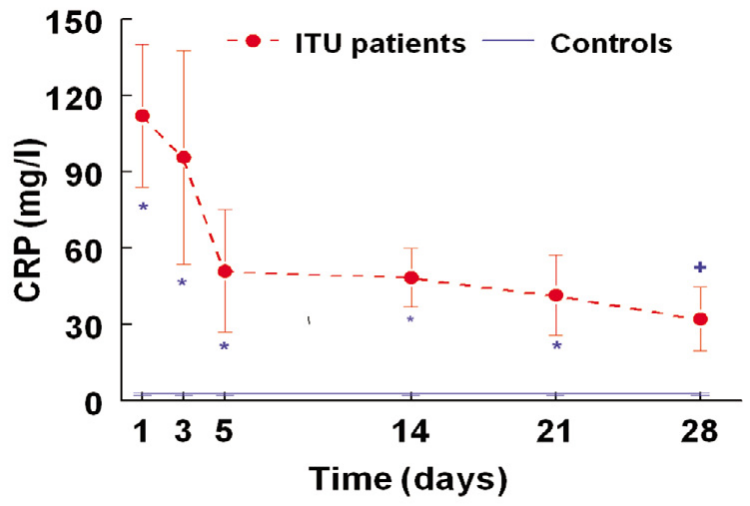
Pattern of plasma C-reactive protein (CRP; mean ± standard error of the mean) during intensive care unit (ICU) stay (n = 8 patients) compared with healthy age and body mass index matched control group (n = 36). Filled circles, ICU patients; solid line, control group; doted line, error bar in control group; *p < 0.05. **p < 0.001 patients versus controls. There was no significant difference between patients and control subjects on day 28. ^+^*p *< 0.05 for patient day 1 versus patient day 28.

Figure 4 shows the degree of acute phase CRP response during ICU stay until day 28. On entering the ICU, patients had significantly higher CRP levels than the control group (99.6 ± 18.4 versus 2.7 ± 0.5 mg/l, *p *< 0.005; Figure 4). There was a statistically significant decrease in CRP levels from the time of enrolment to the time of discharge (99.6 ± 18.4 versus 25.8 ± 10.4 mg/l, *p *= 0.001; Figure 4), but there was still clear evidence of an acute phase response.

### Column chromatography

Gel permeation chromatography demonstrated ghrelin and PYY immunoreactivity eluted at the same position as synthetic ghrelin or PYY (data not shown).

### Markers of appetite and nutritional status

On entering the ICU, patients had significantly lower albumin (16.3 ± 1.4 versus 37.4 ± 1.0 g/l, *p *< 0.005), total protein (45.6 ± 2.6 versus 70.5 ± 1.0 g/l, *p *< 0.005), and haemoglobin (10.0 ± 1.1 g/dl versus 14.1 ± 0.2, *p *< 0.005) than the control group. Evaluating appetite using a VAS suggested that ICU survivors felt less sensation of hunger after discharge from the ICU (24.7 ± 7.4 versus 40.9 ± 4.8 mm, *p *= 0.04), higher nausea (27.3 ± 9.2 versus 5.9 ± 1.4 mm, *p *= 0.03), and higher satiety (43.3 ± 13.3 versus 16.5 ± 2.3 mm, *p *= 0.04) compared with control volunteers. Mean daily energy intake after discharge from the ICU was significantly lower in patients compared to the healthy control subjects (873.4 ± 215.7 versus 1687.9 ± 40.4 kcal, *p *= 0.007).

Markers of appetite and nutritional status changed during stay (from admission into the ICU until discharge) as follows: weight decreased (78.4 ± 0.7 versus 68.1 ± 5.2 kg, *p *= 0.03, n = 7), MAC decreased (28.8 ± 1.6 versus 26.7 ± .0.7 cm, not significant), and triceps skinfold thickness decreased (14.8 ± 4.1 versus 13.9 ± 4.6 mm, not significant). Using the VAS, patients' appetite increased significantly from the time of discharge from ICU to the last week of stay (24.7 ± 7.4 versus 48.3 ± 11.5 mm, *p *< 0.001). Mean daily energy intake on week 4 was significantly lower than estimated energy requirements (873.4 ± 215.7 versus 1687.0 ± 40.4 kcal, *p *= 0.007). Compared to at admission, patients had significantly higher albumin (23.3 ± 1.5 versus 16.3 ± 1.4 g/l, *p *< 0.005), total protein (61.5 ± 3.2 versus 45.6 ± 2.6 g/l, *p *< 0.005), and haemoglobin (11.2 ± 0.4 versus10.0 ± 1.1 g/dl, *p *< 0.05) on discharge. However, these levels on discharge still did not reach healthy control subjects' levels (albumin, 23.3 ± 1.5 versus 37.4 ± 1.0 g/l, *p *< 0.005; total protein, 61.5 ± 3.2 versus 70.5 ± 1.0 g/l, *p *< 0.005; and haemoglobin, 11.2 ± 0.4 versus 14.1 ± 0.2 g/dl, *p *< 0.005).

### Correlations

APACHE II scores and day one PYY levels were positively correlated (r = 0.5, *p *= 0.05 (1-tailed)) and negatively correlated with day one ghrelin (r = -0.3, *p *> 0.05). Percentage increase in ghrelin during the stay was negatively correlated with percentage change in PYY (r = -0.4, *p *> 0.05). Decrease in CRP was positively correlated with decrease in PYY (r = 0.2, *p *> 0.05) and negatively with decrease in ghrelin (r = -0.35, *p *> 0.05). A positive association was observed between patients' food intake at week four and percentage increase in ghrelin (from week 1 to week 4; r = 0.9, *p *< 0.05 (1-tailed)) and there was a negative correlation between food intake and percentage decrease in PYY (r = -0.6, *p *> 0.05). There was a negative correlation between APACHE II score and appetite on day 14 (r = -0.5, *p *= 0.1 (1-tailed)), day 21 (r = -0.7, *p *< 0.05 (1-tailed)) and day 28 (r = -0.8, *p *< 0.1 (1-tailed)).

## Discussion

Anthropometric data pointed to a deterioration in the nutritional status of patients during their stay in hospital, with a decline in body weight, MAC, and albumin and total protein levels. This is consistent with the finding of Giner and colleagues [3], who studied 129 patients admitted to the ICU and followed them until discharge. Nutrition assessment of our patients suggested that their nutritional status was poor prior to admission to the unit, declined further during their stay in the ICU, and malnutrition continued to be a persistent problem during remaining hospital stay. Serial measurements of both mid-upper arm circumference and muscle thickness, using ultrasound, were made by Reid and colleagues [20] in 50 critically ill patients. Muscle wasting was identified in 96% of the patients.

Compared to healthy subjects, our patients exhibited a significantly lower level of ghrelin and a higher level of PYY during their stay in the ICU and afterwards. Both levels gradually returned to normal as they recovered during the further three weeks of their stay. Several previous studies have also suggested that ghrelin and PYY levels are related to appetite and nutritional status. Sturm and colleagues [21] found that plasma ghrelin concentrations were higher in undernourished than well-nourished groups. English and colleagues [22] and Rodrigues Ayala and colleagues [23] found plasma ghrelin levels correlated negatively with BMI. Research also indicates abnormal dynamic levels of PYY and ghrelin in anorexia and bulimia nervosa [24,25].

We observed lower ghrelin levels and higher PYY levels than in a healthy control group. PYY decreases and Ghrelin levels increase over the period of stay in hospital and, as the patients' medical condition improves, their appetite and food intake increase. Recent work by our group on patients with cardiac cachexia caused by severe pulmonary hypertension showed an exaggerated and early PYY response to a test meal when compared to control subjects [11].

It is of interest that in all four patients who had renal failure we found high levels of ghrelin and PYY. Their abnormally high levels of PYY and ghrelin compared to our other patients confirm previous studies that have suggested that the kidney is an important site for clearance and/or degradation of PYY and ghrelin [19,23], although how this observation is reflected in nutritional status is currently unclear.

We found a negative correlation between ghrelin and PYY. A 49% increase in ghrelin was observed from week one to four corresponding to a 45% decrease in PYY. Also, food intake was positively associated with percentage increase in ghrelin and negatively with decrease in PYY. This is consistent with other studies of human subjects [7-10]. A human volunteer study by Batterham and colleagues [10] showed that PYY infusion reduced plasma levels of ghrelin significantly in both lean and obese subjects. This suppression of ghrelin by PYY infusion may add to the anorexigenic effects of PYY. Recent findings suggest that ghrelin has a role in the regulation of meal initiation [7].

In our patients, APACHE II scores were correlated positively with PYY and negatively with ghrelin and appetite during recovery. There might be an association between severity of illnesses and PYY and ghrelin patterns.

This study had methodological limitations. The study group represents a typical heterogeneous ICU population with the associated problems of variability of results. It seems clear, however, that there are consistent abnormalities in PYY and ghrelin levels in ICU patients. Furthermore, the use of nasogastric or parenteral nutrition precluded the accurate assessment of spontaneous food intake and appetite for some patients, thus reducing sample size for some statistical analyses. Sample size was also reduced because four patients had renal failure, which excluded them from the study and four patients died during the study. Nevertheless, the main aim was to investigate the pattern of ghrelin and PYY in very sick patients in hospital and statistically significant results have been established in this report. In spite of these limitations and weaknesses, observations from this study suggest a possible relationship between ghrelin and PYY nutritional intake and nutritional status; consistent with previous studies using PYY and ghrelin infusion or testing pre- and post-prandial levels in normal volunteers [7,8,10].

A particular area of interest is the possible effects of the route of feeding on PYY and ghrelin in ICU patients. In our study, however, three methods of feeding were used in the sample patients (parenteral, nasogastric enteral and oral feeding) and the sample size is too small to draw any conclusions.

Although this is a pilot study on a small number of patients, it raises the intriguing possibility that appetite regulatory peptide levels may be changed by critical illness, thus contributing to continuing nutritional deficits. Further studies are required to establish the role of ghrelin and PYY in acute illness.

This pilot study raises a number of key questions. Why are the levels of these gut hormones altered in critical illness? What is the impact of methods of feeding on these hormones? Does constant feeding have an impact on PYY and ghrelin? What is the effect of drugs which impairs gastrointestinal function? What impact does abdominal pathology or reduced splanchnic circulation have on gut endocrine mechanism? A final intriguing question is whether exogenous ghrelin administration would have any beneficial effect on restoring appetite?

## Conclusion

The nutritional status of patients admitted to the ICU decline over the course of their stay in hospital. Results from our study show high levels of PYY and low levels of ghrelin compared to healthy controls. There may be a relationship between the level of these gut hormones and nutritional intake.

## Key messages

• Recent evidence suggests that gut released peptides, such as ghrelin and PYY regulate the initiation and termination of meals and could play a role in the altered eating behaviour of sick patients.

• This is the first study to report PYY and ghrelin concentrations in critically ill patients.

• We found high levels of PYY and low levels of gherlin compared to healthy controls.

• There appears to be a relationship between concentrations of these pepides and change in nutritional intake and nutritional status over length of stay.

## Abbreviations

APACHE = Acute physiology and Chronic Health Evaluation; BMI = body mass index; CRP = C-reactive protein; ICU = intensive therapy unit/intensive care unit; MAC = muscle arm circumference; PYY/PYY_3–36 _= peptide YY or peptide tyrosine tyrosine; SEM = standard error of mean; VAS = visual analogue scale.

## Competing interests

The authors declare that they have no competing interests.

## Authors' contributions

GF, AB, SJB and MN were responsible for designing the study. JO and LW were responsible for recruiting patients at the ICUs of Hammersmith and Charing Cross Hospitals, respectively. GF, AB and SJB conceived the study, and supervised the data collection and analysis. SR B, MG and MP contributed for radioimmunoassays, column chromatography, and interpretation of ghrelin and PYY results. MN was responsible for taking blood, data collections and analysis, doing radioimmunoassays, column chromatography assays, statistical analysis and manuscript preparation. All authors read and approved the final manuscript.

## Figures and Tables

**Table 1:** Diagnosis of ICU patients

No.	Age (years)	Feeding method	Target energy in ICU	APACHE II	ROD	LOS (ICU)	LOS (total)	Diagnosis	Reason for admission to ICU
1^a^	68	Enteral-NG	1,800	27	0.302	12	48	MVR, hypotensive perioperatively	Haemodynamic, respiratory and renal instability
2^b^	57	Enteral-NG	1,800	14	0.106	14	42	Cardiac tamponad following heart surgery	Haemodynamic and respiratory disruption
3^b^	72	Oral	2,000	23	0.489	4	45	Chest pain and collapse	Haemodynamic and respiratory disruption
4^b^	75	Enteral-NG	1,800	25	0.561	4	26	Biliary sepsis	Haemodynamic instability
5^a^	77	Oral	1,700	23	0.46	9	17	COPD and pneumonia	Haemodynamic instability
6^b^	35	TPN	2,000	16	0.256	16	60	Acute pancreatitis	Haemodynamic and respiratory instability
7^c^	18	Enteral-NG	1,900	16	0.256	8	10	Congenital abnormality in lung vessels	Haemodynamic and respiratory disruption
8^b,c^	82	Enteral-NG	1,800	20	0.517	25	47	Sigmoid vulvulous, large bowl resection	Haemodynamic and respiratory disruption
9^b^	73	TPN	1,700	15	0.126	14	25	Post relaparatomy following pancreatectomy	Haemodynamic and respiratory disruption
10	66	Oral	1,800	13	0.227	6	9	Angioplasty-post insertion of stent	Haemodynamic instability
11^a,c^	64	Enteral-NG	1,800	12	0.07	30	81	Post-emergency laparotomy	Renal instability
12^a^	64	Enteral-NG	1,900	22	0.45	10	84	Post-emergency laparotomy	Haemodynamic and respiratory disruption
13^b^	43	Enteral-NG	2,000	13	0.143	30	76	Global cerebral damage following hypoglycaemia	Hypoglycaemic coma
14^c^	43	Enteral-NG	2,265	12	0.239	13	20	Craniotomy for intracerebral bleeding	Mental deterioration due to haemorrhage in cerebellum
15^b^	80	Enteral-NG	1,800	35	0.823	10	45	Diabetic coma-NIDDM	Diabetic come, collapsed at home
16	40	Oral	1,900	17	0.649	2	10	Sepsis (leukaemia)	General deterioration and confusion

^a^Patients with renal failure who were dialysis dependent. ^b^Patients were considered for ghrelin and peptide YY statistics. ^c^Patients died during stay in hospital. APACHE, Acute Physiology and Chronic Health Evaluation; COPD, chronic obstructive pulmonary disease; ICU, intensive care unit; LOS, length of stay; MVR, mitral valve prolapse; NIDDM, non-insulin-dependent diabetes mellitus; NG, nasogastric; ROD, risk of death; TPN, total parenteral nutrition.

**Table 2:** Demographic details of intensive care unit patients and control subjects

	ICU patients	Control subjects	*P *value
Subjects (n)	16	36	
Age (years)	60 ± 5	54 ± 3	NS
BMI (kg/m^2^)	28.1 ± 1.7	25.8 ± 0.8	NS
Deceased	4		

BMI, body mass index; ICU, intensive care unit; NS, not significant.

**Table 3:** Initial and final plasma concentrations of ghrelin and peptide YY of intensive care unit patients

Ghrelin (pmol/l)		PYY (pmol/l)	
Day 1	Day 28 ± 3	Day 1	Day 28 ± 3

442.4	494.0	22.1	6.4
282.7	516.5	71.6	48.1
43.6	381.4	29.5	19.2
571.2	567.7	22.8	43.6
600.3	743.9	6.1	10.4
126.7	1,136.8^a^	^b^	^b^
212.7	378.8	61.2	11.4
103.1	232.5	7.0	16.3
297.8 ± 76.3	556.5 ± 98.6^c^	31.5 ± 9.6	22.2 ± 6.3^d^

^a^Discharged home on day 21. ^b^Assay failure. ^c^*P *< 0.05. ^d^*P *= 0.17. Values are mean ± standard error of the mean.
